# Auditory ERPs to Stimulus Deviance in an Awake Chimpanzee (*Pan troglodytes*): Towards Hominid Cognitive Neurosciences

**DOI:** 10.1371/journal.pone.0001442

**Published:** 2008-01-16

**Authors:** Ari Ueno, Satoshi Hirata, Kohki Fuwa, Keiko Sugama, Kiyo Kusunoki, Goh Matsuda, Hirokata Fukushima, Kazuo Hiraki, Masaki Tomonaga, Toshikazu Hasegawa

**Affiliations:** 1 The University of Tokyo, Tokyo, Japan; 2 Great Ape Research Institute, Hayashibara Biochemical Laboratories, Tamano, Okayama, Japan; 3 Primate Research Institute, Kyoto University, Inuyama, Aichi, Japan; University of Minnesota, United States of America

## Abstract

**Background:**

For decades, the chimpanzee, phylogenetically closest to humans, has been analyzed intensively in comparative cognitive studies. Other than the accumulation of behavioral data, the neural basis for cognitive processing in the chimpanzee remains to be clarified. To increase our knowledge on the evolutionary and neural basis of human cognition, comparative neurophysiological studies exploring endogenous neural activities in the awake state are needed. However, to date, such studies have rarely been reported in non-human hominid species, due to the practical difficulties in conducting non-invasive measurements on awake individuals.

**Methodology/Principal Findings:**

We measured auditory event-related potentials (ERPs) of a fully awake chimpanzee, with reference to a well-documented component of human studies, namely mismatch negativity (MMN). In response to infrequent, deviant tones that were delivered in a uniform sound stream, a comparable ERP component could be detected as negative deflections in early latencies.

**Conclusions/Significance:**

The present study reports the MMN-like component in a chimpanzee for the first time. In human studies, various ERP components, including MMN, are well-documented indicators of cognitive and neural processing. The results of the present study validate the use of non-invasive ERP measurements for studies on cognitive and neural processing in chimpanzees, and open the way for future studies comparing endogenous neural activities between humans and chimpanzees. This signifies an essential step in hominid cognitive neurosciences.

## Introduction

Chimpanzees, phylogenetically closest to humans, have been studied intensively for comparative cognitive and evolutionary perspectives. Various aspects of cognition have been investigated through dozens of field and laboratory studies, generally using behavioral responses as indices. Other than behavioral indicators, physiological ones such as heart rate [1]–[6], and brain and skin temperature [7] are also included to reflect the subject's internal state. Working with physiological indicators will lead to increasing our understanding of chimpanzee behavior and processes controlling it. Although these measures serve to complement what we know so far, they can only provide a limited amount of information about the neural basis for cognitive processing. In addition to accumulating more human neurophysiological data, it is important to continue investigating the neural activity of chimpanzees. The practical difficulties of conducting non-invasive measurements on awake individuals have certainly restricted the progress in this field.

In human studies, various techniques such as electroencephalogram (EEG), positron emission tomography (PET), and functional magnetic resonance imaging (fMRI) have been introduced to explore underlying neural activities for perceptual, motor and cognitive processing. Event-related potential (ERP), a transient pattern in surface-recorded EEG, is another method that is broadly applied to adult and infant studies; the advantages are temporal resolution and, especially, convenience of measurement. Compared to other techniques, during measurement, ERP is more tolerant of the subject's physical movements; therefore, it is more suitable for subjects whose movements are difficult to regulate. Various ERP components, such as mismatch negativity (MMN), are well documented in humans and are considered to be good indicators of cognitive and neural processing. In chimpanzees, ERP seems to be one of the most applicable and useful tools to non-invasively investigate cognitive and neural processing during an awake state.

In chimpanzees, only two ERP studies have ever been reported. One of them [8] focused on visual evoked potentials, while another [9] focused on auditory ones. [8] measured visual evoked potentials elicited by stroboscopic flash stimuli in three infant chimpanzees (3–7 weeks of age), one infant gorilla (11-weeks of age) and two juvenile chimpanzees (3 and 3.5 years), which were lightly sedated for the measurement. Though only two scalp positions (Oz and Fz) were adopted, they emphasized the similarity of their ERP patterns with those of human. In another study, [9] measured ERPs for various auditory stimuli, such as two kinds of pure tones presented in an oddball paradigm and a call of the subject's name; a 6.5-year old juvenile chimpanzee was lightly sedated during the measurement. For infrequent or informative stimuli, they reported that the late positive component was predominant in the fronto-central area, and argued its consistency with the P3 component that is observed in human. To explore the endogenous neural activities, however, the arousal state of the subject needs to be of concern. In adult humans, patterns of ERP components have been reported to differ depending on the subject's arousal state [10]. Indeed, one of the experimental designs in [9] was identical to the condition typically observed in humans for detecting the MMN component; nevertheless, the MMN-like component was not observed. The participant chimpanzee was lightly sedated with droperidol and ketamine during the measurement. In adult humans, it has been reported that MMN cannot be clearly obtained or attenuated during drowsiness, stage-2 sleep [10], [11], [12], or under ketamine infusion [13]. To further investigate endogenous neural activities in chimpanzees, ERP measurements on fully awake participants are greatly needed.

In this present study, we report ERP values from an awake chimpanzee and especially focus on this well-documented component of human studies, where it is well known that the brain responds differently to infrequent, physically deviant tones compared to frequent tones. Such an ERP component, called mismatch negativity (MMN), has been robustly observed in many precedent studies (for reviews, see ref. [14], [15]). MMN, typically, has a negative deflection (100–250 ms latency from onset of stimulus) over frontal and central scalp positions; it is elicited by any physical change of tones, such as frequency, duration and intensity, in a uniform sound stream. A component similar to MMN that is observed in humans has also been reported in other animals, including macaque (*Macaca fascicularis*: ref. [16], via invasive way), but not in chimpanzee.

We obtained ERP values from a fully awake chimpanzee, while presenting a sound stream of two kinds of pure tones differing in occurrence probabilities (oddball design). We discuss the different neural responses to standard and deviant tones. This is the first study that reports ERPs from a fully awake chimpanzee.

## Methods

### Participant Chimpanzee

Mizuki, a female chimpanzee (*Pan troglodytes*), participated in the ERP measurements. She lives in the Great Ape Research Institute, Hayashibara Biochemical Laboratories, Inc., Okayama, Japan, with other group members (two males, two females, and an infant). She was raised by human caregivers from a few days after birth, but since she arrived at the Great Ape Research Institute when she was two years and one month old, she has spent the majority of her daily time with other chimpanzees outside and inside various compounds. Meals consisted of various vegetables, fruits and primate chows at least three times a day. At the time of experimentation, Mizuki was 9-years old and had undergone other kinds of behavioral cognitive experiments [17], [18].

We took approximately six months to gradually habituate the subject to the experimental device and procedure, and throughout this habituation period she exhibited no negative responses. Care and use of chimpanzee adhered to the “Guide for the Care and Use of Laboratory Animals” of Hayashibara Biochemical Laboratories, Inc., and the research protocol was approved by the Animal Welfare and Animal Care Committee of the University of Tokyo and Hayashibara Biochemical Laboratories, Inc.

### Stimuli

Three kinds of pure sine-wave tones were used for the two-stimulus oddball paradigm. The three tones differed physically in their pitches, having frequencies of 500 Hz, 1500 Hz and 2000 Hz, respectively. These tones, with a length of 100 ms, including 10 ms rise and fall, were generated by audition software (Adobe Audition ver.1.0). The frequencies of these tones were within the audible range of the chimpanzee, and the pitch differences among the tones were larger than the discrimination threshold for each frequency [19]. Therefore, it was strongly suggested that Mizuki readily discriminated the stimulus tones that were used. Sound stimuli were presented through two speakers (BOSE Media Mate II) placed 2 meters away from the subject. The sound intensity measured at the subject position was approximately 80 dB HL on average.

### Procedures

The ERP measurement was conducted in one session, repeated twice consecutively, under each of four conditions combining standard and deviant stimulus tones (Table 1). Within a single session, two tones were assigned to either standard (frequent) or deviant (infrequent) stimuli, respectively, and presented in random order with 0.2 probability of deviant stimuli and 700 ms stimulus onset asynchrony. The subject participated in one recording session per day. At least one week of interval was introduced between measurements when the condition was changed. The whole measurement was completed within 50 days. The chimpanzee was introduced from an outside compound to an experimental room, where the ERP measurements were conducted. The experimental room was covered with concrete walls and the subject sat on a concrete platform, fully awake, during recordings. An experimenter, one of her caregivers, stood in front of her in order to keep her still during the recordings. Each session consisted of 5–7 blocks with 100–200 trials for each block. Between blocks, she was given a rest time, which allowed her to move her body and to receive fruit rewards.

**Table 1:** Combination of stimulus tones for each condition.

stimulus type	condition			
	Condition 1	Condition 2	Condition 3	Condition 4
standard	500 Hz	500 Hz	1500 Hz	2000Hz
deviant	1500 Hz	2000 Hz	500 Hz	500 Hz

### ERP recording and analysis

The EEG was recorded from Ag/AgCl electrodes attached to five scalp positions (Fz, Cz, Pz, C3, C4), according to the International 10–20 system for humans. All electrodes were referenced to a left ear with FPz serving as ground. The electrodes were filled with Quick GEL and impedances were kept below six kΩ. Signals were amplified by NuAmp-40 and processed by Acquire 4.3 software (NeuroScan Inc.) with 1 ms sample time. In the offline analysis, a 0.1–30 Hz band-pass filter (24 dB/oct) was applied. Epochs of 700-ms duration, starting 100 ms prior to stimulus onset, were obtained. These epochs were baseline-corrected with respect to the mean amplitude over the 100-ms pre-stimulus period. Epochs that exceeded ±50 µV were excluded from statistical analysis described below. The number of epochs accepted for the analysis of standard and deviant stimuli, respectively, was as follows: 756 and 182 in Condition 1, 829 and 217 in Condition 2, 806 and 202 in Condition 3, and 890 and 227 in Condition 4.

Statistical analysis was performed to detect the difference between the waveforms for standard and deviant stimuli. The difference waves were obtained for each condition separately and average negative-peak-amplitudes of respective difference waves were calculated for each electrode within 100 ms to 250 ms after stimulus onset, which is the time-window of MMN that is commonly observed in humans. Among the five electrodes, the electrode having the largest value in average negative-peak-amplitudes was detected, and the negative peak latency of that electrode was used for the following calculation. The average negative-peak-amplitude within 100–250 ms after stimulus onset was largest at Cz in Condition 1, and Fz in Condition 2, 3, 4. The average negative-peak-latencies at those electrodes were 208ms, 125ms, 184ms and 180ms for Condition 1, 2, 3 and 4, respectively. During the ±50 ms time window surrounding the latencies, mean amplitudes were calculated for each epoch. The values were fed into a 3-way analysis of variance (ANOVA) with factors of condition (Condition 1∼4), stimulus type (standard vs. deviant) and electrode (Fz, Cz, Pz, C3, C4). Statistical significance was assessed with a p-value criterion less than 0.001.

## Results

**Figure 1:**
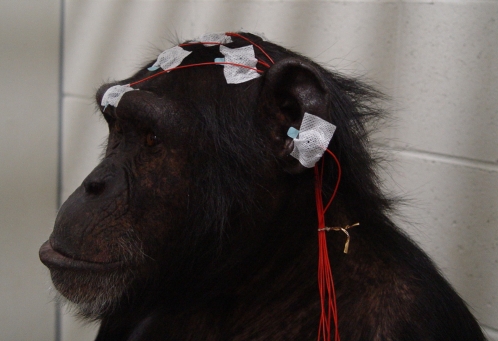
The participant chimpanzee, Mizuki, during ERP measurement.

**Figure 2:**
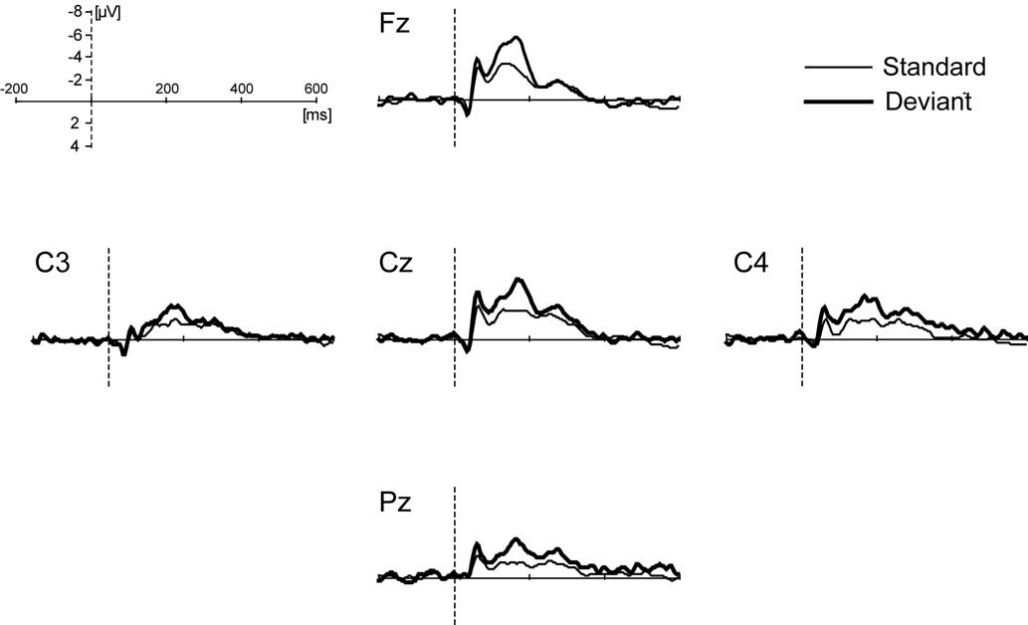
Grand-average waveforms over all conditions for each scalp position. The polarity of the waveforms is plotted with negative values upward.

ERP measurements were conducted in a chimpanzee during a fully awake state (Figure 1). Average amplitudes, within 100 ms window around the negative-peak-latencies, are presented separately in Table 2 for each condition, stimulus type, and scalp position. Statistical analysis revealed significance in the type of stimulus (*F* (1, 20535) = 86.699, *P*<0.0001) and electrode used (*F* (4, 20535) = 20.310, *P*<0.0001), but there were no differences detected when comparing condition (*F* (3, 20535) = 2.658, *P* = 0.047). Figure 2 represents the average waveforms for all conditions at the respective scalp positions. Within the time window adopted, the mean negative amplitude for deviant stimuli was significantly greater than that for standard stimuli. Overall, the average amplitudes tended to be large in the frontal and central areas. However, no interaction was detected for the combination of stimulus type and electrode (*F* (4, 20535) = 0.943, *P* = 0.438), nor for any other combination of variances (condition×stimulus type: *F* (3, 20535) = 1.673, *P* = 0.171; condition×electrode: *F* (12, 20535) = 1.250, *P* = 0.242; condition×stimulus type×electrode: *F* (12, 20535) = 0.474, *P* = 0.931).

**Table 2:** Mean amplitudes (*SD*) of standard- and deviant-waves for each condition and scalp position.

	Condition1		Condition 2		Condition 3		Condition 4	
	amplitude (µV)		amplitude (µV)		amplitude (µV)		amplitude (µV)	
	std	dev	std	dev	std	dev	std	dev
Fz	−1.4 (11.1)	−3.0 (11.7)	−2.3 (8.8)	−3.7 (9.6)	−1.8 (9.7)	−4.6 (9.9)	−2.6 (9.9)	−4.0 (10.5)
Cz	−1.9 (9.5)	−4.2 (9.7)	−2.0 (8.7)	−3.4 (8.3)	−2.2 (9.1)	−3.7 (9.3)	−2.9 (9.2)	−3.9 (9.0)
Pz	−1.4 (11.1)	−2.4 (9.2)	−0.8 (7.7)	−1.9 (7.1)	−0.4 (8.1)	−1.4 (8.6)	−1.2 (8.2)	−1.8 (7.7)
C3	−1.3 (5.8)	−2.3 (6.4)	−0.8 (4.6)	−1.8 (4.6)	−1.4 (5.4)	−2.4 (5.9)	−1.5 (6.4)	−2.4 (6.7)
C4	−1.5 (9.6)	−3.9 (9.8)	−0.9 (7.4)	−1.9 (7.6)	−1.4 (8.2)	−2.5 (7.9)	−2.1 (8.5)	−2.9 (8.0)

*Note*. Mean amplitudes are indicated with *SD* values in parenthesis. std: values for standard stimuli; dev: values for deviant stimuli.

## Discussion

We measured ERPs from a fully awake chimpanzee by presenting a sound stream consisting of two kinds of pure tones that differed in their occurrence probabilities. The results clearly and consistently indicated that infrequent, physically deviant tones elicited larger negative components than frequent tones did, irrespective of the combination of stimulus tones. In humans, such a negative component is commonly reported as MMN (mismatch negativity). For a consistent experimental paradigm, scalp positions and latency were measured where a negative component was observed, and it is assumed that the present component in the chimpanzee was similar to the human MMN. The present study reports event-related potentials in a fully awake chimpanzee for the first time.

MMN-like components in this study were observed over all scalp sites, although the overall signals tended to be greater in the front-central area. In human adults, MMN is generally elicited in greater amplitudes at the frontal and central area, rather than in the parietal area [20]. Compared to human adults, the brain and skull size are smaller in chimpanzees. Distribution of musculature on the skull and its thickness also differs [21]. Such anatomical discrepancies might contribute to the difference in component distribution observed in the present study. A broader MMN scalp distribution has also been reported in human infants and children, although the reason for this remains to be shown [22].

In humans and other animals, the MMN amplitude and latency have been shown to be dependent on the magnitude of stimulus deviance [14]; a large variation between standard and deviant stimuli evokes a larger MMN amplitude. In contrast, the current study did not result in a significant difference between conditions of smaller and larger deviance size. In previous studies, the stimulus deviance was performed within 500Hz in frequency, which is much less than the present study [e.g. 14]. Even in the smaller deviance size paradigm utilized for the present study, the deviance size between standard and deviant stimulus tones had a frequency of 1000Hz. It is possible that the variation in the smaller deviance size paradigm was sufficient to elicit the large negative component. A ceiling effect could be responsible for the lack of a significant effect in deviance size for this study. In order to further prove that the observed negative component is consistent with MMN, more detailed investigations will be required to examine MMN characteristics, such as presentation rate, size of deviance, and deviance probability dependence. These points are beyond the scope of the present study, but should be issues to be investigated in the future.

In chimpanzee, only one study has reported ERPs elicited by auditory stimuli [9]. Similar to our study, they presented two kinds of pure tones to a subject in an oddball design. They reported a late positive component in response to infrequent stimuli that was predominant in the front-central area, rather than the MMN-like component observed in the present study, and argued its consistency with the P3 component in human. Procedural differences could be one of the reasons for varying results between the previous and present study, although the details of the procedure were not fully described in [9]. For example, the occurrence probability of deviant stimuli was relatively high and the number of exposure to deviant stimuli seemed to be larger in the present study compared to the precedent study. In humans, the P3 component, which is predominantly observed in the front-central area of the oddball paradigm, is reported to attenuate with repeated exposure to deviant stimuli [23]. More importantly, arousal states of the participants differed substantially between the two studies. Our chimpanzee was fully awake during whole recordings, while the participant in the precedent study was sedated, although lightly enough to be kept conscious during the task. In human adults, it has been reported that MMN cannot be clearly obtained or attenuated during drowsiness, stage-2 sleep [10]–[12], or under ketamine infusion [13]. Though human infants are known to produce MMN during all stages of sleep, sleep plays a much different role in infants compared to adults [22]; perhaps sleep also plays a much different role in the 9-year-old chimpanzee. Overall, patterns of ERPs, including MMN and P3 components, have been reported to differ depending on the participant's arousal states [10]. It is also possible that cognitive and neural processing of auditory stimuli somehow differs between human and chimpanzee, and this would lead to differences in detection of some ERP components such as P3. To further investigate the endogenous neural activities in chimpanzees, ERP measurements on fully awake participants are greatly needed.

Neural activities, recorded via noninvasive techniques, have rarely been reported in hominid species other than human [8], [9]; however, results of the present study elucidate the utility of ERP measurements for exploring noninvasive endogenous neural activities in the chimpanzee. These results will hopefully stimulate future studies to compare endogenous neural activities between humans and chimpanzees, and will signify an essential step in hominid cognitive neurosciences.

## References

[1] Berntson GG, Boysen ST, Bauer H, Torello MW (1990). Conspecific screams and laughter: Cardiac and behavioral responses of infant chimpanzees.. Dev Psychobiol.

[2] Berntson GG, Boysen ST (1989). Specificity of the cardiac response to conspecific vocalizations in chimpanzees (*Pan troglodytes*).. Behav Neurosci.

[3] Berntson GG, Boysen ST, Rovee-Collier C, Lipsitt L (1990). Cardiac indices of cognition in infants, children, and chimpanzees.. Advances in Infancy Research Vol. 6.

[4] Boysen ST, Berntson GG (1986). Cardiac correlates of individual recognition in the chimpanzee (*Pan troglodytes*).. J Comp Psychol.

[5] Boysen ST, Berntson GG (1989). Conspecific recognition in the chimpanzee: Cardiac indices of significant others.. J Comp Psychol.

[6] Parr L (2001). Cognitive and physiological markers of emotional awareness in chimpanzees (*Pan troglodytes*).. Anim Cogn.

[7] Parr L, Hopkins WD (2000). Brain temperature asymmetries and emotional perception in chimpanzees, *Pan trogldytes*.. Physiol Behav.

[8] Boysen ST, Berntson GG (1985). Visual evoked potentials in the great apes.. Clin Neurophysiol.

[9] Berntson GG, Boysen ST, Torello MW (1993). Vocal perception: brain event-related potentials in a chimpanzee.. Dev Psychobiol.

[10] Winter O, Kok A, Kenemans JL, Elton M (1995). Auditory event-related potentials to deviant stimuli during drowsiness and stage 2 sleep.. Clin Neurophysiol.

[11] Paavilainen P, Cammann R, Alho K, Reinikainen K, Sam M (1987). Event-related potentials to pitch change in an auditory stimulus sequence during sleep.. Suppl Clinical Neurophysiol.

[12] Sallinen M, Kaartinen J, Lyytinen H (1994). Is the appearance of mismatch negativity during stage 2 sleep related to the elicitation of K-complex?. Clin Neurophysiol.

[13] Umbricht D, Schmid LL, Koller RL, Vollenweider FX, Hell D (2000). Ketamine-induced deficits in auditory and visual context-dependent processing in health volunteers: implications for models of cognitive deficits in schizophrenia.. Arch Gen Psychiatry.

[14] Näätänen R (1995). The mismatch negativity: a powerful tool for cognitive neuroscience.. Ear Hear.

[15] Näätänen R, Paavilainen H, Titinen F, Jian G, Alho K (1993). Attention and mismatch negativity.. Psychophysiol.

[16] Javitt DC, Schroeder CE, Steinschneider M, Arezzo JC, Vaughan HG (1992). Demonstration of mismatch negativity in the monkey.. Clin Neurophysiol.

[17] Hirata S, Fuwa K (2007). Chimpanzees (*Pan troglodytes*) learn to act with other individuals in a cooperative task.. Primates.

[18] Idani G, Hirata S, Washburn DA (2006). Studies at the Great Ape Research Institute, Hayashibara.. Primate perspectives on behavior and cognition.

[19] Kojima S (1990). Comparison of auditory functions in the chimpanzee and human.. Folia Primatol.

[20] Alho K (1995). Cerebral generators of mismatch negativity (MMN) and its magnetic counterparts (MMNm) elicited by sound changes.. Ear Hear.

[21] Burrows AM, Waller BM, Parr LA, Bonar CJ (2006). Muscles of facial expression in the chimpanzee (*Pan troglodytes*): descriptive, comparative and phylogenetic contexts.. J Anat.

[22] Cheour M, Leppänen PHT, Kraus N (2000). Mismatch negativity (MMN) as a tool for investigating auditory discrimination and sensory memory in infants and children.. Clin Neurophysiol.

[23] Friedman D, Cycowicz YM, Gaeta H (2001). The novelty P3: an event-related brain potential (ERP) sign of the brain's evaluation of novelty.. Neurosci Biobehav Rev.

